# Supplementary material for: Splice-Junction-Based Mapping of Alternative Isoforms in the Human Proteome
Source: Cell Rep. Author manuscript; Available in PMC 2020 Jan 15. (PMC6961840; doi:10.1016/j.celrep.2019.11.026)
Supplement: 3 [file NIHMS1546469-supplement-3.zip › DF2/PXD0010154/Lung-299-P61978-IIPTLEEYQHYKGSDFDCELR.pdf]

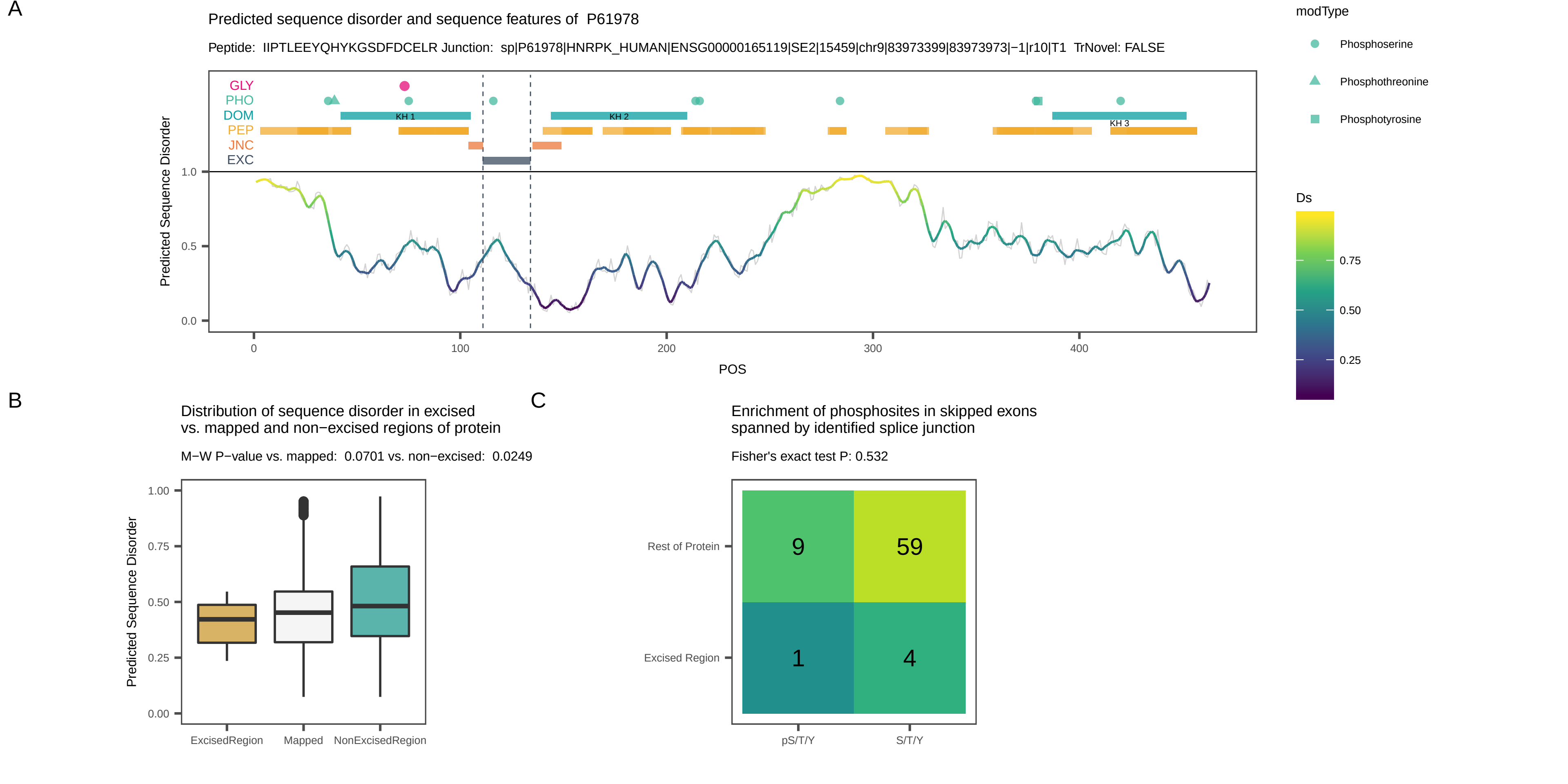
A
Predicted sequence disorder and sequence features of P61978
Peptide: IIPTLEEYQHYKGSDFDCELR Junction: sp|P61978|HNRPK_HUMAN|ENSG00000165119|SE2|15459|chr9|83973399|83973973|−1|r10|T1 TrNovel: FALSE
GLY
PHO
DOM
PEP
JNC
EXC
KH 1
KH 2
KH 3
Predicted Sequence Disorder
1.0
0.5
0.0
0
100
200
300
400
POS
modType
Phosphoserine
Phosphothreonine
Phosphotyrosine
Ds
0.75
0.50
0.25
B
Distribution of sequence disorder in excised
vs. mapped and non−excised regions of protein
M−W P−value vs. mapped: 0.0701 vs. non−excised: 0.0249
Predicted Sequence Disorder
1.00
0.75
0.50
0.25
0.00
ExcisedRegion
Mapped
NonExcisedRegion
C
Enrichment of phosphosites in skipped exons
spanned by identified splice junction
Fisher's exact test P: 0.532
Rest of Protein
Excised Region
9
59
1
4
pS/T/Y
S/T/Y
